# The Immature Stages and Shelter Building Behavior of *Falgo Jeconia Ombra* Evans, 1955 in eastern Ecuador (Lepidoptera: Hesperiidae: Hesperiinae)

**DOI:** 10.1673/031.009.3301

**Published:** 2009-06-02

**Authors:** Harold F. Greeney, Andrew D. Warren

**Affiliations:** ^1^ Yanayacu Biological Station & Center for Creative Studies, Cosanga, Ecuador c/o 721 Foch y Amazonas, Quito, Ecuador; ^2^ McGuire Center for Lepidoptera and Biodiversity, Florida Museum of Natural History, University of Florida, P.O. Box 112710, Gainesville, Florida 32611; ^2^ Museo de Zoología, Facultad de Ciencias, Universidad Nacional Autónoma de México, Apdo. Postal 70-399, México, D.F. 04510 México

**Keywords:** skipper, bamboo, Poaceae, *Chusquea*, cloud forest, Andes

## Abstract

We describe the immature stages and shelter building behavior of *Falga jeconia ombra* Evans, 1955 from eastern Ecuador. *Chusquea scandens* (Poaceae, Bambusoidea) is the larval food plant. Larvae in all stadia build shelters and forcibly eject frass with the aid of an anal comb. Later instars possess an eversible prothoracic “neck” gland. Larvae are associated with moving water.

## Introduction

After a long period of neglect since the early work of Moss (1949), the immature biology of Neotropical skippers (Hesperiidae) has been the subject of renewed interest (e.g. Cock 1991, 1996, 1998, 2000, 2001, 2003; Young 1991, 1994; Burns and Janzen 1999, 2001; Greeney and Warren 2003, 2004). In an effort to continue this trend and to contribute to our knowledge of the ecology of poorly known skipper taxa, we present notes on the life history and shelter building behavior of *F. jeconia ombra* Evans, 1955, a member of the rarely encountered Neotropical genus *Falga* Mabille, 1897.

Mabille (1897: 211) described *Falga* to include a single species, *Carystus jeconia* Butler, 1870 (race *mirabilis* Staudinger *in litt*.], from Bolivia. In the most recent review of *Falga* (Evans 1955: 47–49), there were four included species: *scrias* Godman, 1901, *jeconia* (which itself included five subspecies), *farina* Evans, 1955 and *theoclea* (Hewitson, 1870). *Falga scrias*, described from Honduras, was later reported from Costa Rica (one female) and Mexico (one male) (Evans 1955); no further information on its distribution or biology is available. *Falga farina* was described by Evans (1955: 49) based on two males from Cochabamba, Bolivia; no other details on its distribution or biology are known. *Falga theoclea*, described from Ecuador, was known to Evans from six males and one female (Río Pastaza, Ecuador and Chanchamayo, Peru); no other information on this species is available. The final species assigned by Evans to *Falga* is *F. jeconia*, which includes five subspecies: *jacta* Evans, 1955 (four specimens from Río Titaco, Colombia), *jeconia* (five specimens from Venezuela), *ombra* Evans, 1955 (five specimens from Río Pastaza, Ecuador), *odol* Evans, 1955 (2 ♂♂ from Uruhasi, Peru), and *mirabilis* (five specimens from Bolivia). There have been no subsequent investigations into the taxonomic status of these *F. jeconia* subspecies, perhaps due to the paucity of study material. Careful study of these taxa may demonstrate that more than one valid species-level taxon is involved, since many of Evans' subspecies have been elevated to full species status upon subsequent reevaluation (e.g. Burns 1985, Steinhauser 1989, Mielke 1995, Austin and Warren 2002).

All *Falga* species are rare in collections. It was therefore remarkable to find larvae of *Falga jeconia ombra* abundant in the vicinity of the Yanayacu Biological Station (YBS) and Center for Creative Studies. Our ecological notes on this skipper, based on numerous rearings, are presented below.

## Materials and Methods

We carried out all rearing and field investigations at the YBS (00°35.949 S, 77°53.403 W) located in Napo Province, in the Andes of northeastern Ecuador. The study site is located approximately five kilometers west of the town of Cosanga and includes around 2000 hectares of primary cloud forest bordered by cattle pasture and other disturbed habitats [see Greeney et al. (2006) for a more complete description of the study area]. We collected larvae at elevations ranging from 2000 to 2200 m and reared them at the research station, which is located at 2150 m.

We made three large collections of larvae, pupae, and eggs, and located immature stages by walking along small, forested streams, carefully searching on bamboo leaves up to four meters from the edge of the stream. The first collection (12 November 2000) yielded 49 pupae, 39 fifth instars, and 11 fourth instars. On 23 October 2001, we collected 30 eggs, 29 first instars, 14 second instars, 31 third instars, 4 fourth instars and 3 pupae. The third collection (22 November 2001) produced 163 eggs, 53 first instars, 21 second instars, 8 third instars, 17 fourth instars, and 5 fifth instars. Additionally, we made field observations of all stages from September 2002 to December 2007. We removed larvae to the lab and reared them in plastic bags or glass jars, grouped by instar. When individuals molted we moved them to separate containers to determine stadia lengths. We added fresh food plant leaves as needed, and took body-length measurements for each instar on the day before molt.

We made all observations on larval shelter construction and behavior either in the field or with freshly collected larvae still in their shelters. To avoid potential artifacts resulting from a captive environment, our descriptions and discussions exclude shelters constructed in the laboratory. Terminology follows Greeney and Jones (2003). Vouchers of all life stages have been deposited in the private collections of both authors.

## Results

### Adult behavior

Despite the abundance of immature stages in the study area, we observed adult *Falga jeconia* on only six occasions. Five of these observations were of males guarding perches in areas of bright sun along small forest streams, between 10:00 am and 12:00 noon. Each male patrolled a series of four to five perches between 2–3 m above the ground, and periodically chased passing butterflies, as well as small flies and other flying insects. The perches within a male's territory tended to be less than 1 m apart, and males rarely flew more than 5–6 m in pursuit of passing insects. On one occasion, a male periodically fed at flowers of a species of *Erato* DC (Asteraceae) within his territory. Often, during sunny periods, individuals lowered their hind wings in the typical hesperiine basking position (Figure 2k). In February 2002, we observed a female at 14:00 searching for oviposition sites along a small forest stream in bright sun. She flew rapidly from leaf to leaf, landing most frequently on small, thin-bladed leaves, and rapidly drumming her forelegs on the leaf upper surface. We did not observe any oviposition events.

### General immature biology

All of the 477 individuals of all life stages collected in this study were found directly over moving water. When this pattern became evident, we made a concerted effort to search for larvae away from streams, and in particular, directly adjacent to streams. Despite these efforts, and ongoing caterpillar searches at Yanayacu, no larvae of this species have been found away from moving water. We observed all instars forcibly ejecting frass and found no frass accumulation inside larval shelters. Although not quantified, it appeared that the frass ejection capabilities of *F. jeconia ombra* larvae are weak compared to the abilities of other skippers (Greeney pers. obs.). We reared a total of 43 fourth or fifth instars to adults, and observed no parasitism.

### Egg (Figures 1 a–1 b)
n = 200+; approx. 1 mm diameter; development time > 13 days

**Figure 1.**
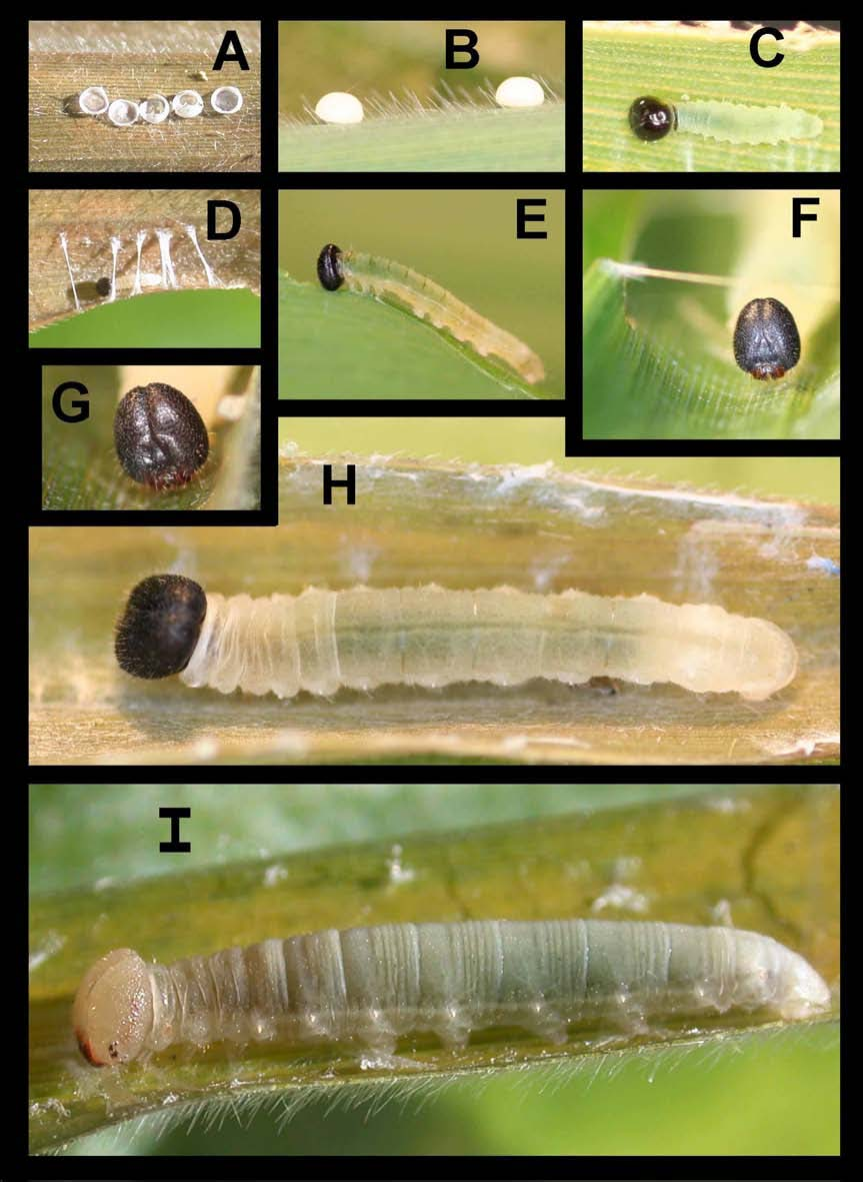
Immature stages of *Falga jeconia ombra* at Yanayacu Biological Station, Napo Province, 2100 m, Ecuador: a) complete clutch of hatched eggs showing variation in the amount of chorion eaten by emerging larvae; b) lateral view of two eggs; c) recently hatched first instar which has already begun to feed; d) first instar, prior to feeding, beginning to construct its shelter at the edge of a bamboo leaf; e) late first instar, showing the change in general body shape from earlier in instar; f) close up of fourth instar head; g) detail of fourth instar head; h) dorsal view of mid-fourth instar; g) lateral view of mid-fifth instar, note faint brown markings laterally on the head.

Egg flattened, dome-shaped, appearing smooth but with minute pitting visible under dissecting microscope, pale yellow to cream-colored (Figure 1b); eggs of a clutch generally laid in one or two loose rows (Figure 1a); upon emergence, larvae eat a hole through top of egg, consuming up to two-thirds of shell, but never entire shell (Figure 1a); 71 clutches of eggs averaged (±SD) 4.8 ± 2.6 eggs per clutch.

### First instar (Figures 1 c–1 e) n = 80+; body length = 2.0–4.8 mm; development time = 13–14 days

Head round to roundly triangular when viewed from the front, smooth, shining black to dark brown with sparse minute pitting and sparse short pale setae; body at hatching widest at A3, abdominal segments slightly projected laterally (Figure 1c, 1d); later in stadium these projections less noticeable, body more elongate (Figure 1e), nearly parallel-sided but gently tapering anteriorly, anterior abdominal segments only slightly produced laterally; body clear yellow-white with dark green viscera showing dorsally after feeding; entire body with sparse, short, pale setae; thoracic legs weakly sclerotized, same color as body; trachaeoles visible beneath cuticle, forming a thin white lateral line connecting spiracles; spiracles white and slightly projecting outward into tubes with small webs of trachaeoles visible beneath cuticle surrounding spiracles, giving them a pinched appearance; pronotum strongly sclerotized, dark brown, produced laterally, extending to subdorsal area (Figure 1c); anal plate weakly sclerotized and transparent, appearing wrinkled or reticulated, bearing two long, pale setae along posterior margin.

### Second instar
n = 40+; body length = to 7 mm; development time = 8–10 days

Head roundly triangular when viewed from the front, smooth shiny black with fine, web-like sculpturing and sparse short pale setae; body similar to 1st instar but lacking strongly sclerotized pronotum.

### Third instar
n = 40+; body length = to 11.2 mm; development time = 9–11 days

Head and body similar to 2 instar, except setae more golden, fringe around anal plate more dense, and spiracles more prominent and produced outward.

### Fourth instar (Figures 1 f–1 h) n = 38+; body length to 18.1 mm; development time = 12–15 days

Similar to 3rd instar; males with a pair of dull purple, kidney to oval-shaped testes, with cream-colored markings visible through cuticle on either side of midline on A6 and A7.

### Fifth instar (Figures 1i, 2a–2f) n = 70+; body length = to 29.8 mm; development time = 24–28 days

**Figure 2.**
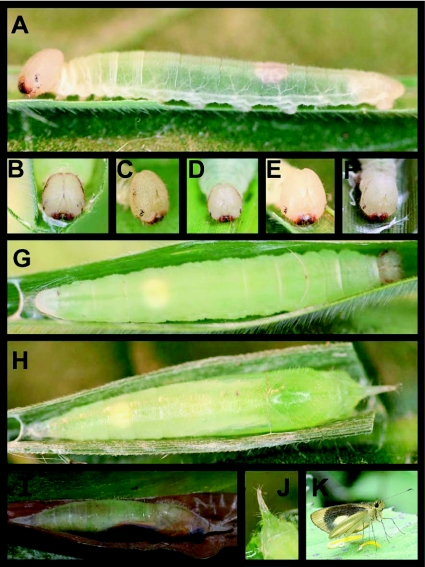
Immature stages of *Falga jeconia ombra* at Yanayacu Biological Station, Napo Province, 2100 m, Ecuador: a) lateral view of late fifth instar, note lack of brown lateral markings on the head; b-f) details of fifth instar head, note variation in the development of brown lateral markings; g) pre-pupa; h) dorsal view of a freshly formed pupa; i) lateral view of a pupa two days prior to adult eclosion; j) detail of anterior projection on head of pupa; k) adult male on a perch in the sun, with hind wings lowered.

Head cream-colored, mouth parts dark reddish brown, stemmata dark brown to black (Figures 2b-2f), some individuals with an indistinct reddish brown wash laterally from stemmata to just below the epicranial crease, this marking highly variable (Figures 1i, 2a-g), roundly rectangular (slightly taller than wide), densely pitted and sparsely covered with short, pale setae; setae slightly longer around stemmata and mouthparts; body transparent whitish, appearing frosted with dark green viscera showing through in a narrow but distinct mid-dorsal stripe from T3 to A10; intersegmental membrane wrinkled, often giving the appearance of yellowish banding; trachaeoles showing as a distinct, thin, white spiracular stripe, spiracles white with a pinched appearance (see description of 1st instar); entire body sparsely covered with short, pale golden setae, longest on anal plate.

### Pre-pupa (Figure 2g)
n = 80+; length = 20–25 mm; development time = 2–4 days

Body of mature fifth instar (pre-pupa) almost entirely translucent lime green.

### Pupa (Figures 2h-2j) n = 90+; length = 20.6–24.5 mm; development time = 21–28 days)

Elongate, widest at A3 or A4 and tapering toward both ends; head bearing a long, thin, anteriorly-directed projection (Figure 2j); proboscis sheath free, extending to A8; entire pupa pale, translucent lime green, cremaster and head projection tinged with pink (Figure 2h), proboscis sheath black at apex; dorsum of head, thorax, abdomen and head projection bearing sparse short pale setae; silk spun prior to pupation bright white, pupa attached to a thick band of silk tied across shelter, with a thin strand of silk across thorax; several days before eclosion eyes turn dark brown, wing pads turn dark brownish orange, head and thorax become dull olive (Figure 2i).

**Figure 3.**
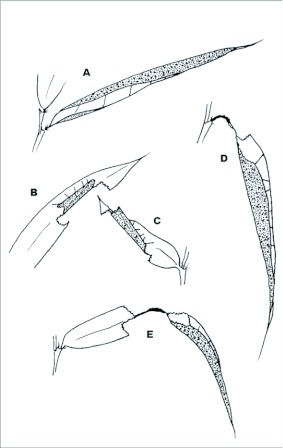
Larval leaf shelters of *Falga jeconia ombra* on *Chusquea* bamboo at Yanayacu Biological Station, Napo Province, 2100 m, Ecuador: a) fourth instar shelter soon after completion; b) first instar shelter after the commencement of feeding; c) second instar shelter twisted and wrapped around the first shelter; d-e) late fourth or fifth instar shelters showing how feeding damage at the base of the leaf allows the shelter to droop from its horizontal position.

### First instar shelter (Figures 1d, 3b, 4a) n = 400+; size = ca. 4–11 mm × 2–4 mm

**Figure 4.**
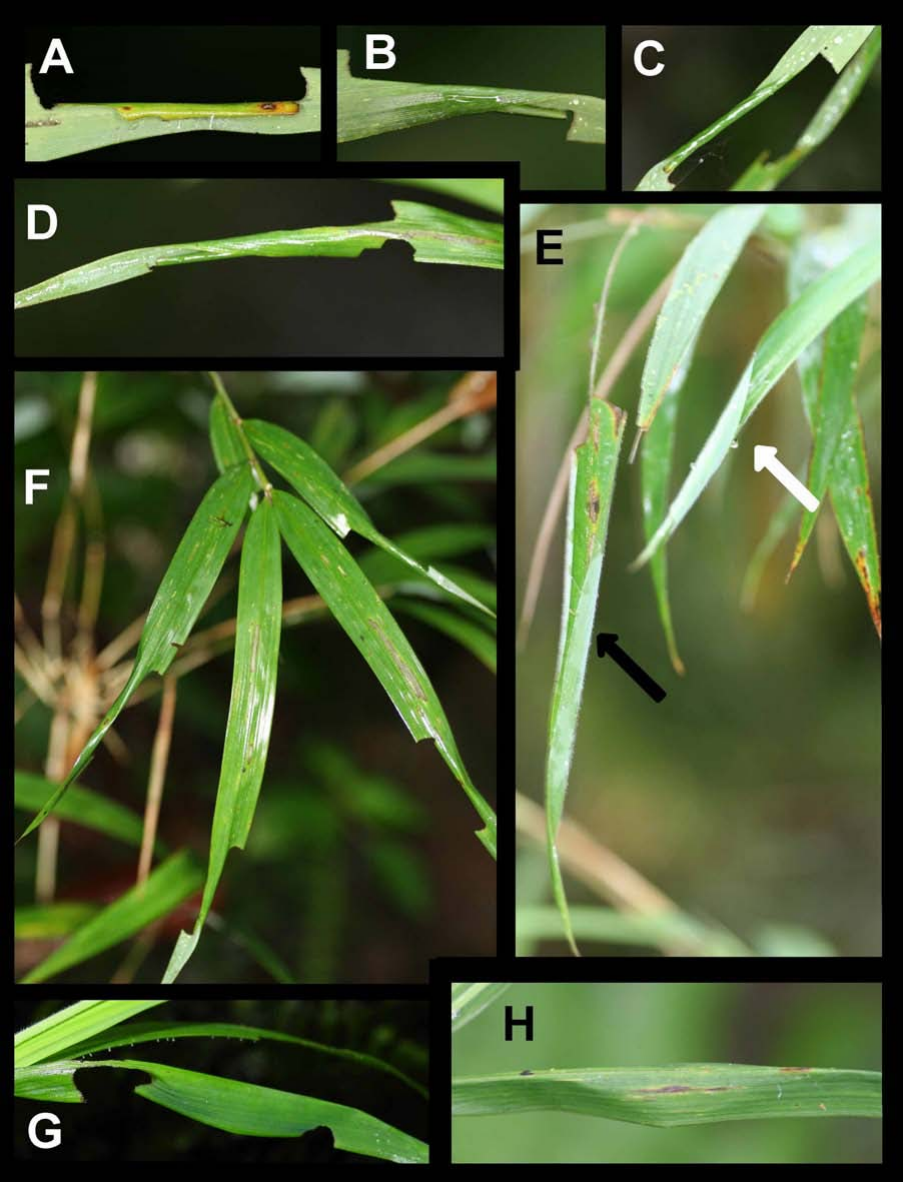
Larval shelters of *Falga jeconia ombra* at Yanayacu Biological Station, Napo Province, 2100 m, Ecuador: a) first instar shelter, note the final, rectangular form; b-d) second instar shelters viewed from various angles; e) shelters built by fourth and fifth instars, the black arrow indicates a shelter where feeding damage has skeletonized the leaf above the shelter, the white arrow indicates a recently built shelter before the commencement of feeding; f) terminal leaves of bamboo food plant showing four second instar leaf shelters; g-h) leaf shelters used for pupation, viewed from above so that the long opening, and thus the pupa, are hidden from view.

First instar shelters are constructed by rolling a small portion of the leaf margin (Figure 1d) either onto the upper or lower surface of the leaf, forming a loose, tubular shelter here termed a “group I, type 2, no-cut fold shelter.” Once the shelter is complete, feeding begins above and below the shelter, and the loose tube is soon tied with silk to form a tight pocket, open at only one end (Figures 3b, 4a). At this stage, the shelter no longer resembles its original form and, if the ontogeny of shelter construction was not known, would be mistakenly termed a “group III, type 9, two-cut unstemmed” shelter. The overall shape is roughly rectangular (Figure 3b).

### Second instar shelter (Figures 3c, 4b-d, f) n = 100+; size = ca. 15–31 mm × 5–15mm

Immediately after or immediately prior to a molt, larvae make an addition to the first instar shelter that involves silking more of the leaf into a tube or flattened pocket around the initial shelter. Often this produces a simple twisting of the leaf around the initial shelter (Figure 4c). Essentially, the first shelter becomes wrapped in additional leaf tissue, providing the protection of two layers of leaf tissue surrounding the larva. Larvae continue to use the first shelter (inside the second) for several days after the second is built, but as they slowly consume the first shelter, they are eventually concealed by the second only. As no additional cuts are made to create the second shelter, it would be termed a “group I, type 2, no-cut fold” shelter (Greeney and Jones 2003. Often the presence of numerous shelters on a host plant is the easiest way to find larvae (Figure 4f).

### Third instar shelter n = 30+

Early third instars were found resting in the shelter made by the second instar. Based on empty shelters found in the field, it appears that late third instars build a third shelter on a separate leaf from the previous two. These shelters are as described for fourth instars; the molt to fourth instar occurred inside them.

### Fourth instar shelter (Figure 3a, d, e, 4e) n = 30+; size = ca. 50–120 mm × 5–9mm

Fourth instars encountered in the field build “group I, type 2, no-cut fold” shelters. These consist of an entire leaf rolled into a tube, sealed near the tip of the leaf, usually with a circular opening at the base. Leaf margins were joined with 7–10 strong silk ties, often leaving the part of the shelter at the base of the leaf partially open along the side, especially right after construction (Figures 3a, 4e). Feeding takes place near the base of the leaf while the larva remains mostly inside the shelter. Late in the fourth stadium, feeding damage causes the leaf to sag into a near vertical position as the leaf blade is removed from both sides of the midvein near the base (Figure 3d) or middle of the leaf (Figure 3e). Larvae rest with the head at the opening of the shelter, and, as there is no room to turn around in the shelter, must crawl entirely out of the shelter to eject frass. Judging from empty shelters found in the field, we suspect that some, if not all, fourth instars build a second fourth shelter identical to that described above.

### Fifth instar shelter (Figures 3d, e, 4e) n = 100+; size = ca 30–60 mm × 5–9 mm

Fifth instar shelters are similar to those described for late fourth instars. We do not know whether fifth instars normally build a final shelter, but suspect that some remain in the fourth stadium shelter and continue to eat the base of the leaf, as described for fourth instars. The larvae essentially consume their shelter from the leaf base toward the apex, and some fourth and fifth instars likely build several shelters before reaching maturity.

### Pupal shelter (Figures 2g-i, 4g-h)
n = 60+; size = ca. 50–90 mm × 8–14 mm

Pupae were found in tented leaf shelters, formed by drawing opposite leaf margins together to form a tent, pinched tightly above and below the pupa, and open toward the underside of the leaf (Figures 4g, 4h). The shelter resembles an overturned canoe. While this shelter conceals the pupae when viewed from above, it leaves them exposed from below, most closely resembling a “group 1, type 2, no-cut fold” shelter. Usually, all feeding had ceased by the time this shelter was constructed, and the leaf was relatively unmodified (Figure 4h). Occasionally, however, larvae fed above and below the shelter, which perhaps aided in folding of the leaf (Figure 4g).

## Discussion

The positioning of larvae and egg clutches directly over moving water, in combination with the weak frass-throwing ability of the larvae, suggests that moving water may be important in frass removal. Ejection of frass from the shelter, thus removing it as a potential olfactory cue, has been suggested as a means to reduce parasitism and/or predation (e.g. Weiss 2003). The potential use of moving water for frass removal, in conjunction with decreased frass-throwing ability, deserves further study.

The ontogenetic change in shelter types described here closely parallels that described for the hesperiine *Vettius coryna coryna* (Hewitson, 1866) (Greeney and Warren this issue). However, two major differences are apparent, both involving modifications of the shelter after its initial construction. The first involves the final form of the first larval shelter. In *V. coryna*, feeding damage occurs only at one end of the original leaf curl, giving the final product a roughly triangular shape. In contrast, feeding damage by *F. jeconia* occurs at both ends of the larval shelter, resulting in a roughly rectangular shape. The second difference can be seen in the final larval shelters, which again are of the same basic type but are modified differently. While both shelters hang perpendicular to the natural plane of the leaf, their positioning is accomplished in different ways. In *V. coryna*, the leaf midvein is partially cut and silk is used to bend the leaf tip downward (Greeney and Warren this issue). In *F. jeconia*, vertical positioning of the shelter is accomplished by simply weakening the leaf's structure when the leaf blade is eaten on both sides of the midvein. Different construction processes that produce convergent architectural designs have recently been noted for first instar shelters of *Epargyreus clarus* (Cramer, 1775) and *Urbanus proteus* (Linnaeus, 1758) (Greeney and Sheldon 2008). As shelter architecture for additional species become known, subtle differences such as these should be noted carefully, since they may prove to be phylogenetically informative.

The shelters built by 5th instars of both *V. coryna* and *F. jeconia* are quite similar. As noted above, this upside-down-canoe shaped shelter does not fit well into the larval shelter classification scheme of Greeney and Jones (2003) because it does not “mostly or completely hide the larva (pupa) from view.” Therefore, following the scheme developed by Greeney and Jones (2003), we call this new shelter type a “group I, inverted canoe shelter.”

As noted in the description of 1st instar shelters, their form may change slightly when modified by feeding. This may result in misinterpretation of the shelter types proposed by Greeney and Jones (2003), revealing an important flaw in their classification system. In combination with observations by the first author on additional species, this suggests that the entire process of shelter construction, in addition to the final product, is important for fully understanding shelter form. However, we feel that the system proposed by Greeney and Jones (2003) remains useful for classifying gross shelter types and for discussion of shelter form. We hope that the detailed observations presented here will encourage others to record detailed comparative observations on larval shelters. While these have traditionally been neglected in life history studies, they may ultimately provide a unique and highly useful character system for the Hesperiidae.

## Editor's note

Paper copies of this article will be deposited in the following libraries. Senckenberg Library, Frankfurt Germany; National Museum of Natural History, Paris, France; Field Museum of Natural History, Chicago, Illinois USA; the University of Wisconsin, Madison, USA; the University of Arizona, Tucson, Arizona USA; Smithsonian Institution Libraries, Washington D.C. USA; The Linnean Society, London, England.
